# Bayesian Network-Based Risk Assessment of Single-Phase Grounding Accidents of Power Transmission Lines

**DOI:** 10.3390/ijerph17061841

**Published:** 2020-03-12

**Authors:** Jun Zhang, Haifeng Bian, Huanhuan Zhao, Xuexue Wang, Linlin Zhang, Yiping Bai

**Affiliations:** 1 State Grid Energy Research Institute Co., Ltd., Beijing 102209, China; zhangjun@sgeri.sgcc.com.cn (J.Z.); bianhaifeng@sgeri.sgcc.com.cn (H.B.); 2 School of Emergency Management and Engineering, China University of Mining & Technology, Beijing 100083, China; 1510130127@student.cumtb.edu.cn (X.W.); zqt1910101052g@student.cumtb.edu.cn (L.Z.); 1410120101@student.cumtb.edu.cn (Y.B.)

**Keywords:** risk assessment, bayesian network (BN), bow-tie, power transmission line, single-phase grounding

## Abstract

With the increasing demand for electricity transmission and distribution, single-phase grounding accidents, which cause great economic losses and casualties, have occurred frequently. In this study, a Bayesian network (BN)-based risk assessment model for representing single-phase grounding accidents is proposed to examine accident evolution from causes to potential consequences. The Bayesian network of single-phase grounding accidents includes 21 nodes that take into account the influential factors of environment, management, equipment and human error. The Bow-tie method was employed to build the accident evolution path and then converted to a BN. The BN conditional probability tables are determined with reference to historical accident data and expert opinion obtained by the Delphi method. The probability of a single-phase grounding accident and its potential consequences in normal conditions and three typical accident scenarios are analyzed. We found that “Storm” is the most critical hazard of single-phase grounding, followed by “Aging” and “Icing”. This study could quantitatively evaluate the single-phase grounding accident in multi-hazard coupling scenarios and provide technical support for occupational health and safety management of power transmission lines.

## 1. Introduction

**Figure 1:**
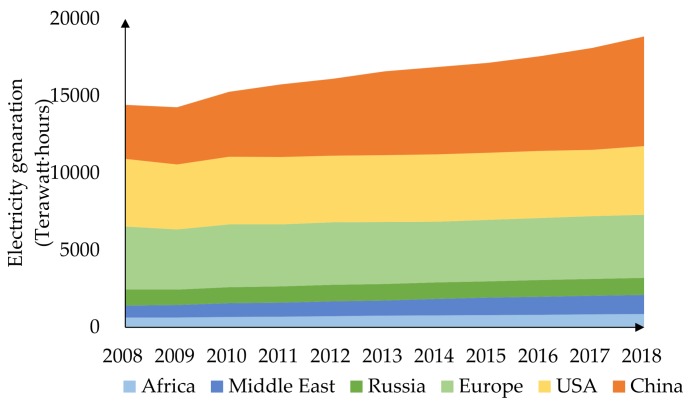
Electricity generations of major countries and regions.

With the rapid urbanization and industrialization of China, the demand for electricity is continually increasing. Figure 1 shows electricity generation in Europe, Russia, USA and China. While electricity generation in other countries and regions has remained stable, China’s electricity generation has continued to grow rapidly and accounts for the world’s largest share [1]. Huge demand for electricity brings both economic benefits and risks. There have been a large number of blackouts around the world, causing great losses, such as the serial blackout in India on July 7, 2012, affecting more than 670 million people, the Brazil blackout in November 10, 2009, affecting 67 million people, the America-Canada blackout on August 15, 2003, affecting around 50 million people, the European blackout on November 4, 2006, affecting more than 10 million people, and the Hunan blackout in February 2008 affecting more than 4 million people [2,3,4,5,6]. A mass blackout is a typical HILP (high impact low probability) accident with extremely low probability. However, minor grid accidents occur frequently, with single-phase grounding being the most common accident of power distribution networks, accounting for around 90% [7]. As the largest grid company in the world, the State Grid Corporation of China supplies power to over 1.1 billion people in 26 provinces in China, and runs more than 1,000,000 km of transmission lines [8]. Transmission over such long distances across multiple environments is difficult and risky. There are several components of transmission systems, such as wires, overhead ground wires, insulators, metal fittings, towers, foundations, etc. Compared with other components, the transmission wire is the most fragile section as it is exposed and easily affected by storms, snow, bird activity, mis-operation and third-party damage. However, causes and consequences of transmission wire damage have not been clearly analyzed yet.

There have been many studies that have focused on operation state evaluation of transmission lines based on Bayesian networks to assess the impact of failure of different sections and support the decision making of the grid operation [9,10,11,12]. Ji et al. [13] used GeNIe, a kind of Bayesian network software, to combine historical state, present state and predicted state as three parent nodes pointed to a comprehensive state to assess the state of UHV transmission lines. Teive et al. [14] proposed a Bayesian network approach to fault diagnosis and prognosis in power transmission systems to identify the most probable cause and the prognosis of potential failures. Shen [15] implemented transmission line fault diagnosis with a dynamic Bayesian network and data obtained by robot inspection to determine risk factors and construct a fault diagnosis model. Moreover, he applied a comparison of simulation outcomes between a dynamic BN and static BN, showing that the former had better performance. Zhu et al. [16] put forward a three-element-oriented model based on a simplified Bayesian network to estimate fault sections of a large transmission power system. Yang and Song [17] proposed a Bayesian Classifier-based Multiple Service Awareness (BC-SA) scheme of Broadband Power Line Communications to schedule resources efficiently. Furthermore, they compared the performance of the Bayesian network, neural network, classical logic expert system and fuzzy logic expert system. Lesmantas and Alzbutas [18] used a hierarchical Bayesian model with North American grid outage data to simulate and assess the reliability of large blackouts. Isamsumang et al. [19] used a dynamic Bayesian network and Markov Chain Monte Carlo inference for health management of on-line systems to improve failure detection and estimation of RUL (Remaining Useful Life). The above studies focused on the state evaluation and fault diagnosis of transmission systems, without analyzing particular grid accidents, nor their causes and potential consequences.

However, only a few studies have been conducted on single-phase grounding accidents, and much less on transmission systems. Velasquez [20] proposed a new method to detect single-phase faults due to atmospheric discharges of long overhead lines, with several practical approaches put forward. Li et al. [21] analyzed the single-phase grounding fault characteristics with a simplified linear model to discuss the effects of imbalance loads and asymmetrical lines. Wang et al. [22] used a Mahal distance discriminant theory-based scheme to improve the stator winding single-phase grounding faults. They chose four factors, including magnitude and direction of leakage current, and magnitude and direction of 0-sequence current in the power transformer terminal, to estimate and evaluate the performance of the discriminant model. Nevertheless, previous studies have been dedicated to single-phase fault detection, characteristic identification and performance evaluation. It seems that we still need a systematic quantitative risk assessment of single-phase grounding accidents of transmission lines that includes the whole process of the accident, the integrated risks and the various losses.

In this study, the Bayesian network was employed to analyze single-phase grounding accidents of power transmission lines. Based on case-studies of typical single-phase grounding accidents with the Bow-tie method, 21 BN nodes were proposed to represent the evolution process of single-phase grounding accidents from causes to consequences quantitatively, including casualties, power supply losses and equipment losses. With the proposed model, the probabilities of different accident scenario consequences and losses are analyzed and evaluated. A sensitivity analysis is applied to identify the key influencing factors, which should be controlled properly to reduce accidents and losses. Then a relevant emergency plan, precautionary measures and regulatory measures will be set, which will avoid accidents or worsening of accident injuries. This study could provide practical support for the occupational safety and sustainable management of power transmission lines.

## 2. Methods

### 2.1. Bayesian Networks (BN)

**Figure 2:**
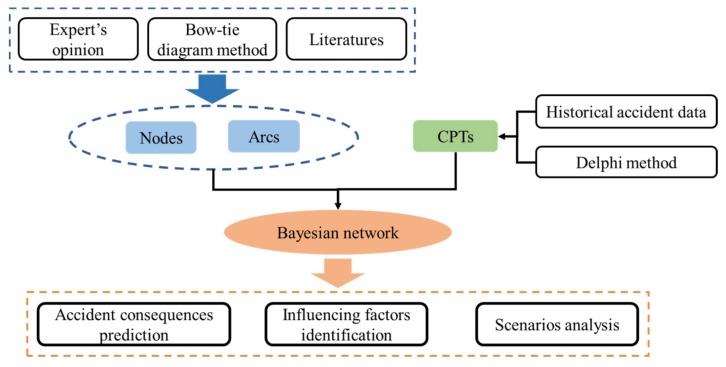
Framework of BN application of this study.

Bayesian networks (BN), also known as Belief Networks or Causal Networks, are a directed acyclic graph (DAG), which is one of the most effective models concerning the fields of uncertain knowledge and inference at present. In this paper, the BN model was described as a graphical network, which indicated the causal relationships between different events and related factors [23]. The BN model can use probability to replace all forms of uncertainty. Furthermore, learning and reasoning can be implemented by probabilistic rules [24]. BN expresses the structure of problems intuitively through the language of graph theory, and the structure of the problem is utilized according to the principle of probability theory [25,26,27]. The BN application in this paper is shown in Figure 2.

There are two nodes, node A and node B, in a directed acyclic graph in the BN, and there is a directed segment from node A to node B. Node A is the parent for node B, and node B is the child for node A. A is the root node if it has no parent node, or A is the leaf node if it has no child node. Node A’s ancestor node includes the parent node and the parent node’s ancestor node. Node A’s descendant node includes node A’s child node and the child node’s descendant node [28,29]. The basic rule of BN evidence theory is given as formula 1:

(1) $$P(V_{i}|V_{i-1},\cdots ,V_{1})=P(V_{i}|Parents(V_{i}))$$

The parent nodes of *V_i_* should include the nodes of *V_1_*, *V_2_*,…*V_i-1_*, which will impact *V_i_*. The order of adding nodes to construct a BN is to add “root cause” nodes first, then add variables directly affected by them, and so on, until reaching “leaf nodes” that have no direct influence on other variables.

The nodes of BN represent the corresponding random variables, and the corresponding random variables of each child node correspond to a conditional probability table. The set of random variables X= {X_1_, X_2_…X_n_} represents the size of the random variable as x={x_1_, x_2_…x_n_} indicates that the parent node set of X_i_ is multiplied by the probability distribution of Pa(Xi) variables to obtain the joint distribution [30], as shown in formula 2:

(2) $$P(X_{i})=\overset{n}{\underset{i=1}{\Pi }}P(x_{i}|pa(X_{i})$$

It is concluded that pa (X_i_) represents the empty set and p(x_i_) represents the prior probability.

### 2.2. Bow-Tie Model

**Figure 3:**
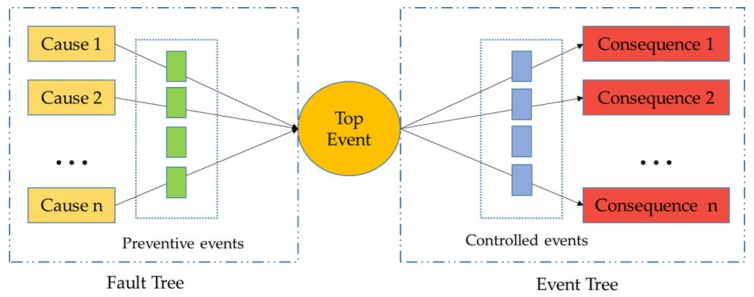
Basic Bow-tie model.

The Bow-tie model is a graphical model that shows the accident evolution path. The key nodes of the accident can be determined, and the preventive control measures used to prevent the occurrence of the accident can be proposed by combining the event tree and the fault tree [31,32]. The middle harmful event that connects the fault tree and the event tree is the top event in the Bow-tie model. As shown in Figure 3, the causes in the fault tree will promote the occurrence of the top event, while the failure to control the top event will finally result in the accidental loss [33]. The advantages of the Bow-tie model are that it is convenient to propose a preventive control measure in the left fault tree and control measures in the number of events on the right [34,35].

### 2.3. Delphi Method

The Delphi method, also called the expert investigation method, is where the organizers of the forecast send questionnaires to the experts, and there is no horizontal contract between the experts, so they express their opinion anonymously. After the statistics and several rounds of feedback, the forecast results gradually converge into a qualitative prediction method [36,37]. 

The opinions of experts are influenced by the information provided by the organizer and by other experts when they give feedback in the Delphi method. However, as long as the organizer’s problem is objective and reasonable, the Delphi method can be regarded as a good qualitative method, and weights can be assigned to authoritative experts in different fields to make the results more reasonable.

**Figure 4:**
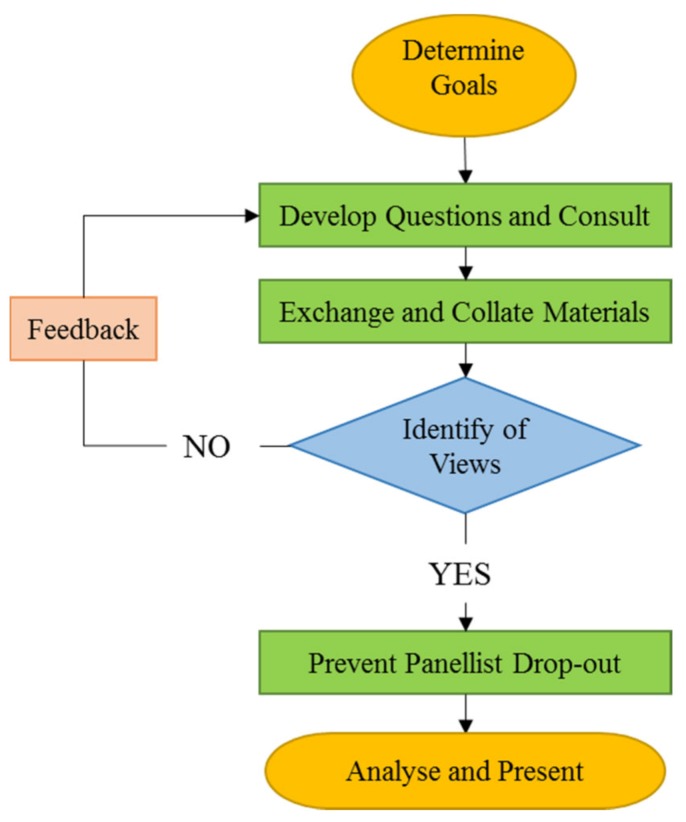
Delphi method flowchart.

As shown in Figure 4, the specific operation of the Delphi method can be based on the following steps of the flow chart, and feedback should be given at least three times in the feedback phase, generally three to five times [38], until the experts reach a consensus.

## 3. Establishing a Bayesian Network

This paper establishes a dynamic Bayesian network for single-phase grounding faults of power distribution lines. There are 21 nodes in the single-phase grounded Bayesian network, including 10 parent nodes and 11 child nodes. In this paper, when defining the accident node, the intervention of the corresponding emergency measures to the accident consequence is not considered. Based on the previous accident statistics and expert judgment methods, the prior probability of the parent node was obtained, and then the probability of the occurrence of each child node was scored by experts using the Delphi method, and the data obtained was input into Netica software (Norsys Software Corp, Vancouver, Canada) to obtain a complete single-phase grounded Bayesian network.

### 3.1. The Structure of the Bayesian Network

The single-phase grounded Bayesian network is transformed from the Bow-tie diagram. As the intermediate node, single-phase grounding connects the accident tree and event tree together to form the Bayesian network of single-phase grounding. Each node is defined as a different state according to the severity of each event. Generally, there are two states for most events, and a few nodes define three states. The specific node state is defined as follows:

(1) Storm (A): The operation of transmission lines is exposed to the natural environment for a long time, and encountering severe windy weather is inevitable. In this paper, this node includes wind only, and not other harsh environmental conditions such as thunderstorms. The influence of strong-wind weather on transmission lines is manifested in two aspects. On the one hand, under the strong driving effect of the wind load, the transmission line will be shaken violently, which will easily cause the transmission line to break and lead to a single-phase ground fault. On the other hand, debris, such as branches, plastics and billboards that break in the storm and float in the air, is likely to have a strong impact on transmission lines, resulting in fracturing and single-phase ground faults. According to the size of the windstorm intensity, three levels are defined: levels 0–6 are “slight”, levels 7–10 are “medium”, and levels 11 and above are “severe”.

(2) Icing (H): When the ice thickness exceeds the design standard for transmission lines (10–30 mm) under frozen natural disaster conditions, deformation of poles and towers, transmission line ground guide faults, single-phase ground faults and damage of the power grid equipment occur.

(3) Lightning strike (B): A lightning strike disaster is one of the main reasons for transmission line faults. After direct lightning strikes the transmission line, it will generate overvoltage and a large number of mechanical, thermal and electromagnetic effects, which will easily cause the transmission line to fuse and lead to a single-phase ground fault. It can also break down the insulation layer of the transmission line, reducing the insulation performance of the equipment and causing equipment damage. If there are less than 10 lightning bolts or the echo intensity is less than 40 dBz in the tower section over 24 h, the lightning strike intensity is defined as “slight”; otherwise, it is defined as “severe”.

(4) Safety training (C): The impact of safety training on single-phase ground faults of transmission lines is mainly manifested by a lack of safety training or poor staff participation in the training, which will lead to a defect of the safety management level, resulting in an insufficient on-site operation ability of front-line workers. The single-phase grounding fault of power transmission lines is affected to some extent. According to the knowledge integrity of safety training and the effect of employees participating in safety training to obtain knowledge, the definition of safety training is divided into two states, “good” and “insufficient”.

(5) Management system (D): The condition of the management system will have a significant impact on the healthy operation of power grid enterprises. According to the actual situation of the power grid management system, if the standard specification of the power grid conforms to the national standard, meets the industry characteristics, has enterprise characteristics and is highly operable, it is defined as “sound”; otherwise, it is “unsound”.

(6) Theft (E): According to the current uneven level of economic development, theft of power transmission lines in rural areas occurs frequently, including power theft and cable theft. This easily leads to single-phase circuit failure of power lines, resulting in varying degrees of power loss. Depending on the actual situation, if a theft has occurred, it is defined as “yes”; otherwise, it is “no”.

(7) Construction damage (G): The increase of large-scale construction activities, such as rock blasting, crane operation and blasting operations, can easily affect the normal operation of the transmission line. For example, the blast wave of a blasting operation may destroy the transmission lines, iron towers and other equipment, resulting in varying degrees of damage to the operation of the power grid. According to whether construction damage occurs, construction damage is defined as “yes” or “no”.

(8) Incorrect operation (F): During transmission line maintenance, the workers, due to their own ideological paralysis and negligence, make a series of operations in violation of safety regulations, which may cause transmission line failure, casualties and other consequences. The error operation is divided into two node states, “yes” and “no”.

(9) Equipment aging (J): Transmission lines, iron towers and poles, which are vulnerable to sun exposure and rain corrosion, are exposed to the natural environment all year round. Thermal oxygen aging occurs on the surface of the equipment, which reduces its insulation performance. According to the actual aging degree of equipment, if it does not affect normal use, it is defined as “slight”; if it causes power supply loss and grid operation failure, it is defined as “severe”.

(10) Design defect (K): As stipulated in the Product Quality Law, the product itself is an unreasonable danger that endangers the safety of human life and other people’s property. There may also be design defects in power grid equipment, such as an unreasonable grid structure that causes transmission line fracture, tower collapse and other faults. According to the actual situation, the design defect can be defined by two states. If the safety performance of the power grid operating equipment does not comply with laws and regulations, or there are security risks, it is defined as “yes”; otherwise, it is defined as “no”.

(11) Environmental causes (L): Generally speaking, the influence of environment on single-phase ground faults of transmission lines refers to severe weather. As the intermediate node, the environment causes the single-phase ground fault, which is mainly evaluated from the status of the three parent nodes. According to the actual operating environment of the transmission line, environmental factors are divided into two states: “good” and “bad”.

(12) Improper management (M): Improper management is a common indirect cause that may lead to low economic benefit of enterprises or cause accident casualties. It is mainly manifested as an incomplete enterprise management system and inadequate safety training. According to the actual situation of the power grid, improper management is defined as “yes” or “no”.

(13) Human error (N): Transmission line failures caused by human factors mainly include theft, construction damage and wrong operation. Human causes can cause transmission line failure, which causes single-phase grounding. Human causes are defined as two node states, “yes” and “no”.

(14) Equipment failure (Q): Equipment failure refers to the failure of equipment due to aging or design defects. The grid equipment failures can be defined as “yes” and “no”.

(15) Single-phase grounding (R): Single-phase grounding is a common fault of transmission lines in power systems. It refers to a three-phase circuit in which one phase is connected to the earth, forming a closed circuit, causing electric shock, fire and other accidents, and eventually leading to economic losses and casualties. According to the actual situation, single-phase ground faults are defined as “yes” and “no”.

(16) Grid instability (S): This refers to the state in which the voltage fluctuation deviates from the normal standard range due to the occurrence of single-phase grounding accidents, but not fire or electric shock accidents. If the range of supply voltage fluctuation is less than 10% of the nominal voltage, it is “slight”; if it is more than 10% of the nominal voltage, it is “serious”.

(17) Electric shock (T): The occurrence of a single-phase ground fault, such as a ground wire break, can easily cause a human or animal electric shock accident. According to the previous single-phase grounding accident cases, electric shock accidents can be defined by two states, “yes” or “no”.

(18) Fire (U): When a single-phase ground fault occurs on the transmission line, the temperature will rise, which ignites flammable objects around the grid frame, causing different fire events. According to the previous single-phase grounding accident cases, fire accidents are defined by two states, “occurring” or “not occurring”.

(19) Power supply losses (V): For power supply losses caused by transmission line failure, we refer to The Regulations on Emergency Handling and Investigation of Power Safety Accidents. If regional power grid load reduction is less than 7%, it is “slight”; if regional power grid load reduction is 7–15%, it is “medium”; and if regional power grid load reduction is more than 15%, it is “severe”.

(20) Casualties (W): The Regulations on Reporting, Investigation and Handling of Production Safety Accidents defines “slight” as less than 3 deaths or less than 10 serious injuries; “medium” is defined as less than 10 deaths or less than 50 serious injuries; and “severe” is defined as 10 or more deaths or more than 50 serious injuries.

(21) Equipment losses (X): For economic loss due to equipment damage caused by transmission line failure, we also refer to the Regulations on Reporting, Investigation and Handling of Production Safety Accidents. If the loss is less than $1 million, it is “slight”; if the loss is more than 1 million yuan and less than 10 million yuan, it is “medium”; and if the economic loss is more than 10 million yuan, it is “severe”.

### 3.2. Conditional Probability Table 

A Bayesian network has an advantage in solving an uncertain situation. Because of the lack of statistics and data, determining the main effects of single-phase grounding and developing accident prediction methods uses the Delphi method to obtain the probability data of each node [39]. Each node is scored according to the experts’ experience in the form of questionnaires, and the average value is finally obtained. The experts who scored for our study had extensive experience with single ground failures and subsequent accidents.

Although it is difficult to evaluate the specific probability value of each node, the probability given by the six experts is very meaningful for us in studying single-phase grounding faults and accidents caused by single-phase grounding. The probability of parent nodes is shown in Table 1.

For instance, the node “Storm” is selected from all the parent nodes. Its probability distribution (87.16%, 12.8%, 0.04%) is scored by experts, which indicates that experts believe the probability is the greatest when the impact of the storm is slight and the probability of a severe storm is very small. It is relatively easy to obtain the evaluation probability of the parent nodes, while the probability of the child node is affected by the probability of the parent node.

Furthermore, “Improper management” is due to the parent nodes, “Safety training” and “Management system”. In Table 2, six experts scored the probability of all the scenarios of the two parent nodes, and the values are the prior probability distribution of “Improper management”. For example, *m_1_(1,2)* means that the first expert considers the probability of the states of “Improper management” to be 1.01 and 98.99 in the case of relatively good training and a sound management system.

Then, according to the consistency test formula, as follows, the expert scoring results are analyzed to determine whether it is necessary to give feedback again for scoring [40].

(3) $$\alpha =\frac{K}{K-1}\left(1-\frac{\sum_{i=1}^{K}\sigma^{2}Y}{\sigma^{2}X}\right)$$

**Figure 5:**
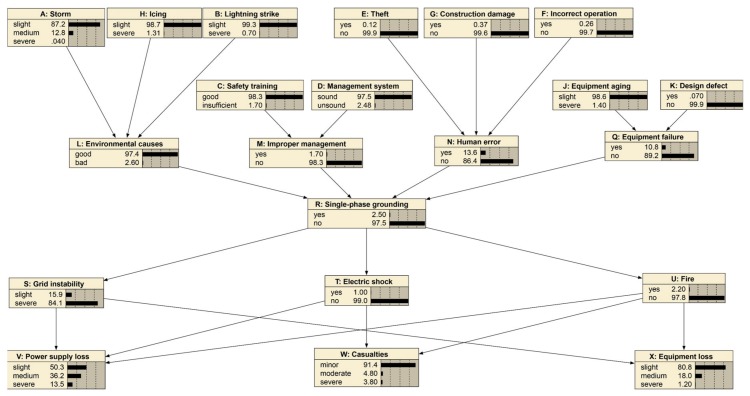
Initial Bayesian network for representing single-phase grounding accidents.

In this formula, “α” stands for the Cronbach’s alpha. “σ^2^X” stands for the variance of the expert’s score results for all scenarios. “σ^2^Y” stands for the variance of the expert’s score results for a particular scenario. “K” stands for the number of questions. If the value of “α” is greater than 0.8, the expert consensus is considered. The Bayesian network is established when the expert opinions on the state of each node reach a consensus as shown in Figure 5.

## 4. Cases Study 

Case analyses that reflect how the Bayesian network can be carried out to analyze different “real-scenario” states are abundantly available. The occurrence and consequence probability of serious event can be predicted by the BN model, which is helpful for proposing preventative measures and formulating an emergency plan. There are three typical cases shown below:

### 4.1. Case 1: Cause of Severe Storm and Severe Strike

**Figure 6:**
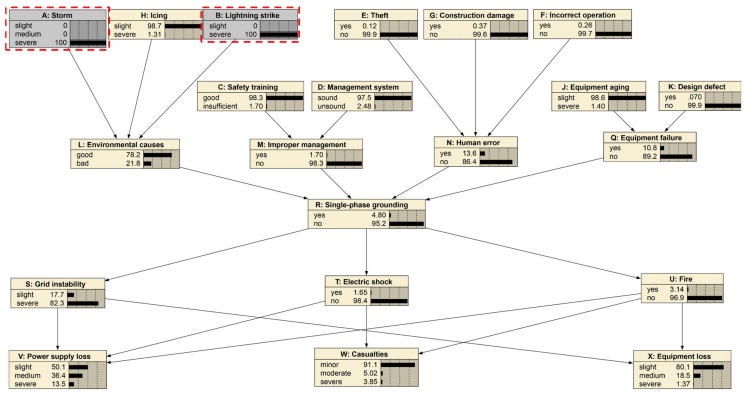
Case study for severe storm and severe strike.

**Figure 7:**
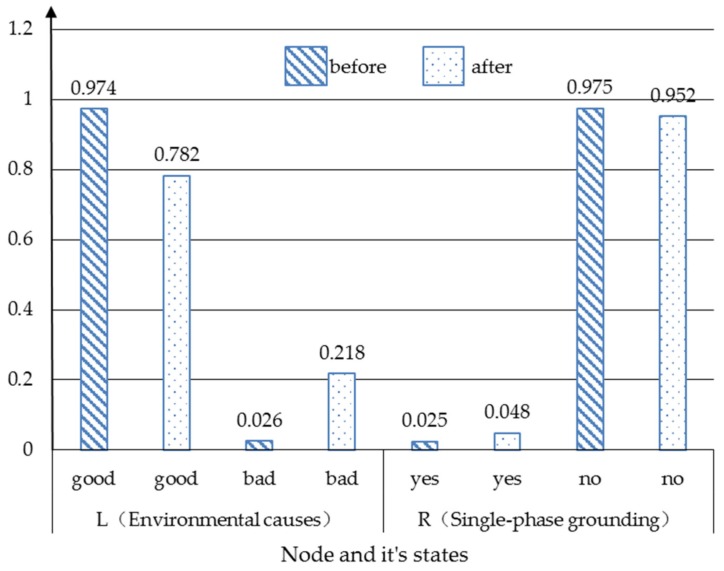
The probability changes before and after the “Environmental cause” and “Single-phase grounding” case.

This case identifies the vital factors of severe “Storm” and severe “Lightning strike”. As given in Figure 5 and Figure 6, the prior probability of “Environmental cause” shows 97.4% as good and 2.6% as bad, and “Single-phase grounding” shows 2.5% as “yes” and 97.5% as “no”. The probabilities will change if the parent nodes’ probabilities change. A typical example is that severe “Storm” and severe “Lightning strike” will cause the probabilities of “Environmental cause” and “Single-phase grounding” to make a difference. When storm and lightning strike is severe, it can be seen from Figure 7 that the probability of good “Environmental cause” will reduce by 19.2% to 78.2%, which means the reverse side will increase by 19.2% to 21.8%. Meanwhile, the probability of “yes” for “Single-phase grounding” will increase by 2.3% to 4.8%, and the probability of “no” will reduce by 2.3% to 95.2%.

This case shows that the environment factors are exceedingly vital for single-phase grounding. In other words, it is necessary to formulate emergency plans and control measures in order to avoid serious events of single-phase grounding.

### 4.2. Case 2: Theft, Incorrect Operation

**Figure 8:**
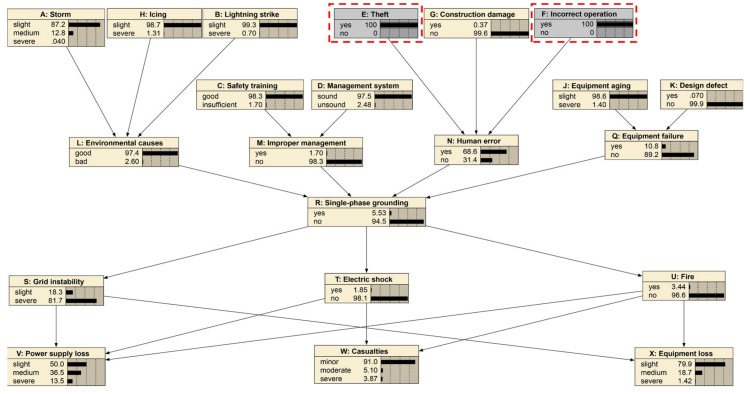
Case study for theft and incorrect operation.

**Figure 9:**
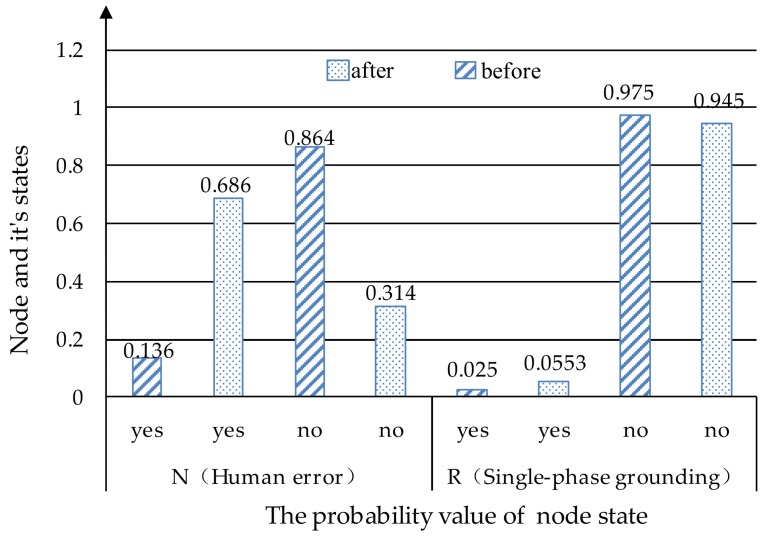
The probability changes before and after the “theft” and “incorrect operation” case.

This case will examine the effects of “Theft” and “Incorrect operation” on “Human error” and “Single-phase grounding”. The Bayesian network is show in Figure 6. In the initial Bayesian network, the probability of human error occurring was 13.6%, and the probability of a single-phase ground fault being “yes” is 2.5%. However, in the case of both “Theft” and “Incorrect operation” occurring, as shown in Figure 8 and Figure 9, the probability of “Human error” became 68.6%, which increased by 55%. The probability of a “single-phase ground” increased by 3.03% to 5.53%. Thus, “Theft” and “Incorrect operation” are important causes of “Human error” and “Single-phase ground”. The security monitoring of power grids should be strengthened, and the theft of power grids and power facilities should be investigated and severely punished to reduce the occurrence of “Theft”. At the same time, it is necessary to strengthen the professional skills of distribution network workers and minimize the mis-operation to ensure the safe operation of the power grid.

### 4.3. Case 3: Storm, Management System, Incorrect operation, Design Defect

**Figure 10:**
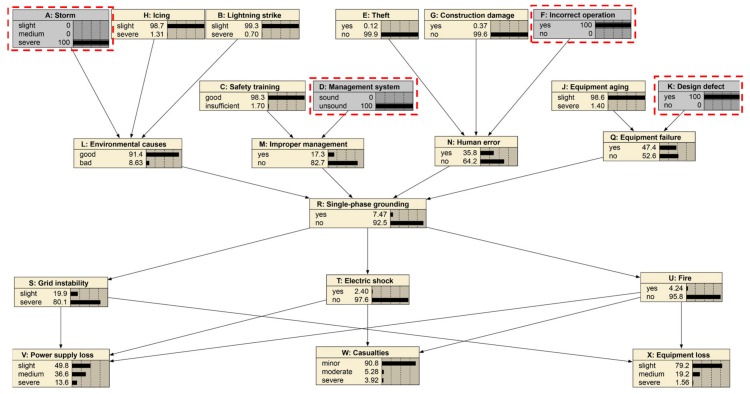
Case study for storm, management system, incorrect operation and design defect.

**Figure 11:**
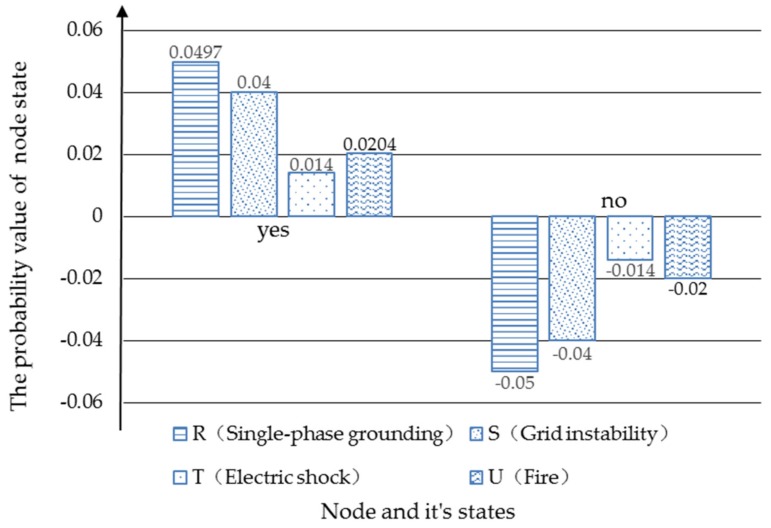
The probability changes before and after “Single-phase grounding”, “Grid instability”, “Electric shock” and “Fire”.

**Figure 12:**
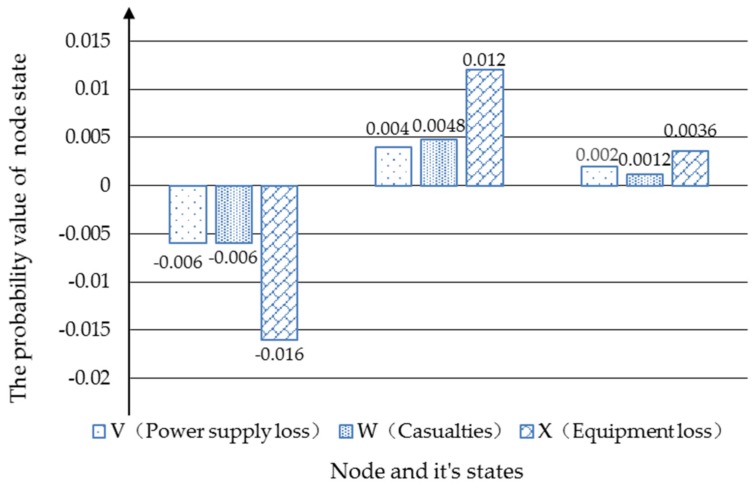
The probabilities change before and after “Power supply loss”, “Casualties” and “Equipment loss”.

When the states of “Storm”, “Management system”, “Incorrect operation” and “Design defect” are “yes”, the probability value of “Single-phase grounding”, “Grid instability”, “Electric shock”, “Fire” and other parents nodes will change. The Bayesian network is shown in Figure 10. As shown in Figure 11 and Figure 12, the probability of “single-phase grounding” becomes 7.47%, increasing by 4.97% after setting the scenario. The probability of “grid instability” increased by 4%, the likelihood of “electric shocks” increased by 1.4% and the probability of a “fire” rose by 2.04 percent.

Under the same scenario setting, the severities of “Power loss”, “Casualties” and “Equipment loss” increased as shown in Figure 12. The medium situation rose by 0.4% and the severe one rose by 0.2%. For “Casualties”, the severe situation rose by 0.12 percent. However, the “slight” state for “Device loss” declined by 0.16%, and “medium” and “severe” rose by 0.12 percent and 0.36 percent, respectively.

## 5. Sensitivity Analysis (SA)

Sensitivity analysis (Shorthand for the “SA”) plays an important role in the process of a Bayesian network. It is a useful method analyzing uncertainty according to the changes of child nodes when different foot nodes (influencing factors) change to find out the most sensitive factors. The key influencing factors are found by sensitivity analysis. Then, the relevant emergency plan, precautionary measures and regulatory measures are set, which avoids accidents or worsening of accident injuries.

### 5.1. SA for “Environmental Causes”, “Improper Management”, “Human Causes” and “Equipment Failure”

**Figure 13:**
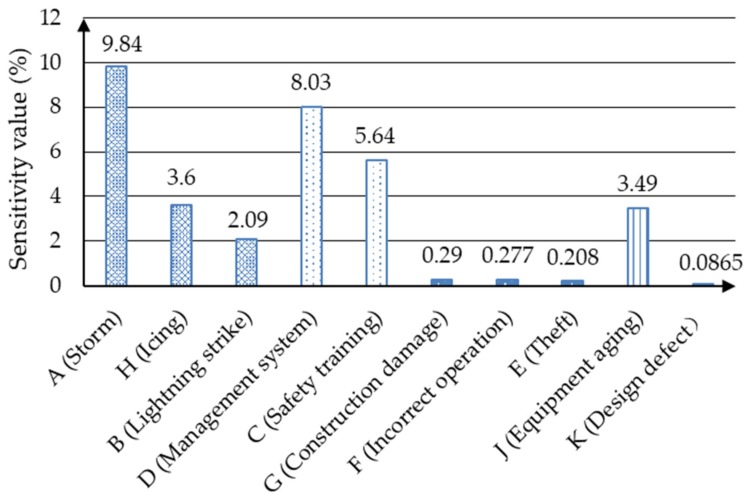
Sensitivity of the root of “Environmental causes”, “Improper management”, “Human causes” and “Equipment failure”.

In this part, a sensitivity analysis is conducted to examine the sensitivity of “Environmental causes”. The results show that the most critical factor on “Environmental causes” is “Storm”, whose sensitivity value is 0.0984. Then, the sensitivity analysis is done to identify the sensitivity of “Improper management”, where the most important factor is “Management system” in the management aspect. The factor that affects the sensitivity analysis of “Human causes” is “Construction damage”, which means it is more crucial than “Theft” and “Incorrect operation”. Finally, the sensitivity of “Equipment failure” is analyzed as shown in Figure 13. “Equipment aging” has a particular impact on “Equipment failure”, whose sensitivity value is 0.0349.

Through this part of the sensitivity analysis results, we can conclude that particular attention should be paid to the storm’s impact in such bad weather conditions. Furthermore, companies also need to develop a sound management system, because it also plays an important role in the management aspect. However, the differences in sensitivity values of various behaviors of personnel are small, which means that all kinds of human error behaviors may cause accidents. In addition, in order to avoid the influence of equipment aging on single-phase grounding accidents, regular professional maintenance and routine inspections are very essential.

### 5.2. SA for “Single-Phase Grounding” 

**Figure 14:**
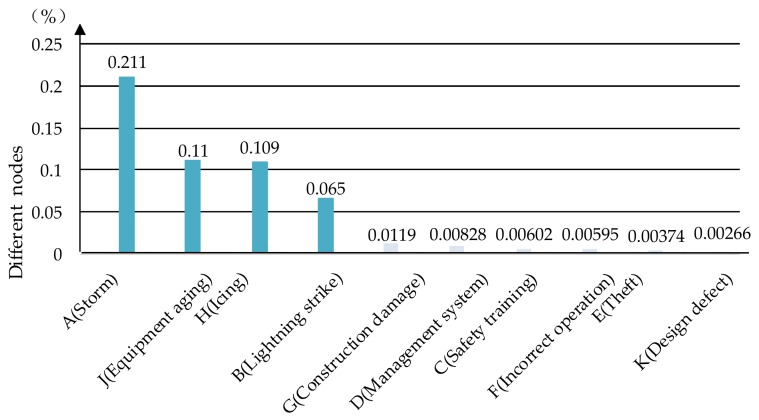
Sensitivity value of nodes for “Single-phase grounding”.

In the last part, the sensitivity analysis is conducted to test the sensitivity of “Single-phase grounding” from the original parent nodes. As seen in Figure 14, the exceedingly vital nodes are “Storm”, “Equipment aging”, “Icing” and “Lightning strike”. Their values are 0.00211, 0.0011, 0.00109 and 0.00065, which means severe weather conditions, such as a storm, icing or lightning strike, and equipment aging are of great significance for the occurrence of single-phase accidents. In severe weather conditions, we should strengthen the early warning and forecast, and make emergency plans for the weather situation to reduce the influence. Furthermore, equipment aging will increase the possibility of single-phase grounding accidents, so regular professional maintenance is necessary. Moreover, the environmental risks may couple with and affect equipment failures, improper management and human causes, which means normally “acceptable” risk will be “unacceptable” in rough environmental conditions such as storms, icing or lightning. In addition, the reduction effect is even more pronounced when more than two kinds of environmental risk exist together.

## 6. Conclusions

In this paper, a Bayesian network for representing single-phase grounding accidents of dynamic power grids is proposed based on the combination with the Bow-tie method. The main contributions and conclusions are summarized as follows.

(1) The Delphi method and expert scoring method were integrated to process the main disaster factors of the single-phase grounding of transmission lines, and finally a dynamic Bayesian network consisting of 21 nodes was established. The initial Bayesian network shows that the probability of single-phase grounding accidents occurring is 2.5% with slight casualties and equipment losses but 13.5% for severe power supply losses and 36.2% for medium power supply losses. For modern society, power supply stability has a growing impact on the economy and society, which needs more attention.

(2) On the basis of the sensitivity analysis, it was discovered that “Storm”, “Equipment aging”, “Icing” and “Lightning strike” are the main influencing factors for “Single-phase grounding”. Moreover, “Storm” is the most critical factor for “Single-phase grounding”, which means that strengthening storm, icing and lightning strike management measures is very necessary. Meanwhile, the environmental risks may couple with other risks and degrade the safety performance of the whole system. Thus, grid safety in extreme environments should have more importance attached, both technically and politically. In addition, the resilient grid needs a flexible power network topology so that the self-healing ability can be enhanced.

(3) As one of the most common and influencing factors, “Equipment aging” plays an important role in single-phase grounding accidents. As everyone knows, professional maintenance and routine inspection are very effective in preventing aging. However, because of the long distance and out-of-town environment, the traditional inspection of transmission lines is hard to perform. Therefore, intelligent perception and robot inspection will be analyzed and applied in the future.

## Figures and Tables

**Table 1:** Probability of parent nodes.

Bayesian Nodes	State of Bayesian Nodes	Probability of Each State (%)
Storm	①slight	87.16
	②medium	12.8
	③severe	0.04
Icing	①slight	98.69
	②severe	1.31
Lightning strike	①slight	99.3
	②severe	0.7
Safety training	①good	98.3
	②insufficient	1.7
Management system	①sound	97.52
	②unsound	2.48
Theft	①yes	0.12
	②no	99.88
Construction damage	①yes	0.37
	②no	99.62
Incorrect operation	①yes	0.26
	②no	99.74
Equipment aging	①slight	98.60
	②severe	1.40
Design defect	①yes	0.07
	②no	99.93

**Table 2:** Prior probability distribution of “Improper management” (%).

BN Nodes		Expert’s Opinion					
Safety training	Management system	*m_1_(1,2)*	*m_2_(1,2)*	*m_3_(1,2)*	*M_4_(1,2)*	*M_5_(1,2)*	*M_6_(1,2)*
good	sound	(1.01, 98.99)	(1.08, 98.92)	(1.05, 98.95)	(0.98, 99.02)	(0.96, 99.04)	(1.04, 98.96)
good	unsound	(16.85, 83.15)	(18.06, 81.94)	(17.50, 82.50)	(16.96, 83.04)	(17.23, 82.77)	(16.84, 83.16)
insufficient	sound	(17.85, 82.15)	(16.96, 83.04)	(18.83, 81.17)	(18.05, 81.95)	(18.15, 81.85)	(16.54, 83.46)
insufficient	unsound	(21.50, 78.50)	(22.05, 77.95)	(21.88, 78.12)	(21.50, 78.50)	(22.10, 77.90)	(22.07, 77.93)
